# Understanding factors influencing care seeking for sick children in Ebonyi and Kogi States, Nigeria

**DOI:** 10.1186/s12889-020-08536-5

**Published:** 2020-05-24

**Authors:** Leanne Dougherty, Kate Gilroy, Abimbola Olayemi, Omitayo Ogesanmola, Felix Ogaga, Chinwe Nweze, Joya Banerjee, Chioma Oduenyi, Michel Pacqué

**Affiliations:** 1 Maternal and Child Survival Program (MCSP), John Snow, Inc. (JSI), 2733 Crystal Dr 4th Floor, Arlington, VA 22202 USA; 2 Health Systems Consult Limited (HSCL), Abuja, Nigeria; 3 MCSP, Jhpiego, Baltimore, MD USA

**Keywords:** Child health, Care seeking, Gender, Nigeria, Pneumonia, Malaria, Diarrhea, Fever

## Abstract

**Background:**

Nigeria has one of the highest child mortality rates in the world, with an estimated 750,000 deaths annually among children under age five. The majority of these deaths are due to pneumonia, malaria, or diarrhea. Many parents do not seek sick-child care from trained, biomedical providers, contributing to this high rate of mortality.

**Methods:**

This qualitative study explores factors enabling or preventing parents from seeking care for sick under-five children in Nigeria’s Kogi and Ebonyi states, including gender-related roles and social norms. Interviews were conducted with parents of sick under-five children and service providers, and focus group discussions were held with community leaders to assess how care-seeking behavior was influenced by four modes from the Colvin et al. conceptual framework for household decision-making and pathways to care. These include (1) caregivers’ recognition and response to illness, (2) seeking advice and negotiating access within the family, (3) making use of community-based treatment options, and (4) accessing biomedical services.

**Results:**

Parents were found to have a general understanding of illness symptoms but did not always attribute illness to biomedical causes. Intra-household decision-making processes were shaped by gender dynamics between men and women, and were found to have great effects on decisions to seek care. Use of traditional medicine and self-treatment were found to be common first steps in treatment before turning to the biomedical care system. Once the decision to seek biomedical care was taken, the route of seeking care varied between seeking care at chemists and then continuing to health facilities or starting with a health facility and then accessing prescriptions from a chemist.

**Conclusion:**

We conclude that care-seeking decisions do not follow a linear process; that intra-household decision-making processes particularly among parents should not be underestimated in addressing sick-child care seeking; and that, given the role of mothers as primary caregivers, their knowledge in illness recognition and agency in care-seeking decision-making, and seeking biomedical care, is deserving of future study.

## Background

Nigeria, the most populous country in Africa, has approximately 32 million children under the age of 5 years. Approximately 750,000 children under five die annually in Nigeria and with a national under-five mortality rate of 109 deaths per 1000 live births, it has one of the highest under-five mortality rates in the world [1, 2]. Pneumonia, malaria and diarrhea are the leading causes of death in the post-natal period representing more than two-thirds of all post-natal child deaths [3, 4]. In addition, these illnesses contribute to high levels of child morbidity, with an estimate of over eight million episodes of clinical pneumonia occurring annually in Nigeria [5]. Despite high levels of morbidity and mortality from pneumonia, malaria, and diarrhea, most families do not seek care from a trained biomedical provider. According to the 2016–17 Nigeria Multiple Indicator Cluster Survey, less than a third of children whose mother reported symptoms of pneumonia, fever, or diarrhea in the 2 weeks prior to the survey received care from a biomedical provider, such as a nurse at the primary health center [6].

Many factors influence if, when and where families seek sick-child care. Poorer families around the world [7, 8] including Nigeria [9–12] are less likely to seek and receive appropriate care and treatment for their sick children. Similarly, mothers who have not attended school have lower rates of appropriate care seeking when compared to those that have attended school [12–15]. Distance to a health facility and financial barriers (both direct costs associated with treatment and indirect costs associated with transportation and lost productivity) often influence families’ care-seeking practices [12, 13, 15]. Not surprisingly, families who live closer to a health facility are more likely to seek care for their children at the facility [10, 15]. In a qualitative study carried out in Nigeria’s Kebbi and Cross River states, the costs of seeking care and mother’s limited access to financial resources to seek care were cited as barriers to seeking care for childhood pneumonia, diarrhea, and malaria [16].

Family relationships, household decision-making patterns, control of household resources, and gender roles can also greatly affect care-seeking practices [13]. Gender, social and cultural norms in Nigeria often dictate that a mother must consult her husband or in-laws prior to seeking care outside the home [16]. Women in Nigeria who are the sole providers of household income or those who have access to their own money are more likely to access maternal and child health services [13, 17, 18]. Secondary analysis of the Nigeria Demographic and Health Survey 2013 data shows that seeking appropriate care for a child with suspected pneumonia increases almost threefold when the decision is made jointly by both parents as opposed to the mother alone [9]. Fathers are responsible for family resources and pay for care and often take decisions related to when and where care is sought [19].

**Fig. 1:**
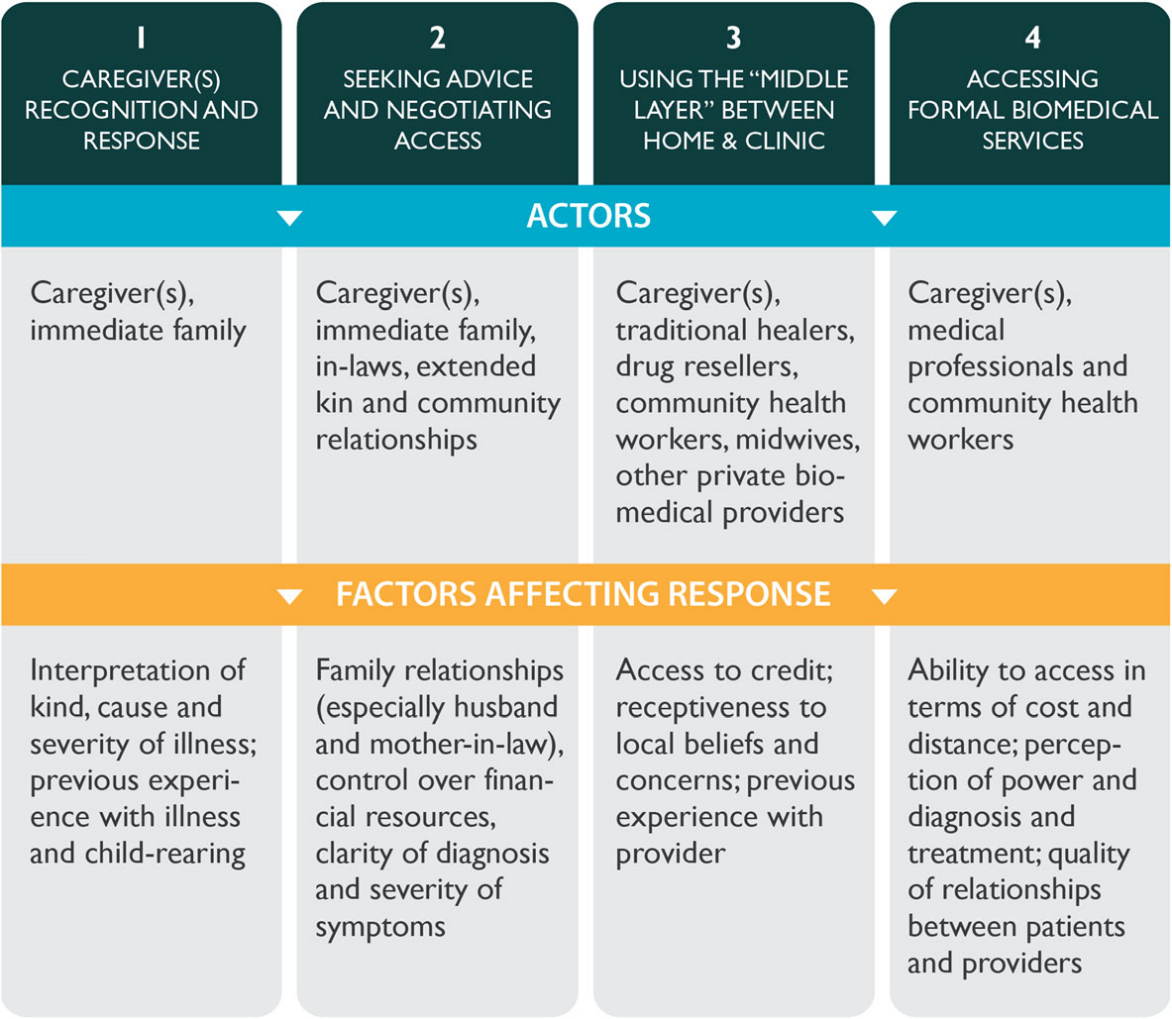
Conceptual framework for sick-child care seeking adapted from Colvin et al

In a 2013 systematic review, Colvin et al. synthesized qualitative evidence on the factors that influence household recognition of illness and response to child diarrhea, pneumonia, and malaria in sub-Saharan Africa. The authors used findings from their review to construct a conceptual framework that aids in understanding household decision-making and pathways to care [13]. Among the studies reviewed, there was a general theme that the courses of action moved from inside to outside the home throughout the course of the illness and operated in four modes: [1] caregiver recognition and response to the illness, [2] seeking advice and negotiating access within the family, [3] making use of community-based treatment options, and [4] accessing formal biomedical services (refers to providers trained in the biological sciences) (see Fig. 1). Individual and contextual factors also influenced treatment choices, and decision-making about treatment was considered a dynamic process [13].

The Maternal and Child Survival Program (MCSP), funded by the United States Agency for International Development, introduced and supported scale-up of high-impact health interventions in priority maternal and child health countries. MCSP worked in Kogi and Ebonyi states with the state Ministries of Health from 2015 to 2018, where few studies have examined care seeking for child illness. The majority of studies examining care seeking for sick children have been undertaken in northern Nigeria and Cross River State, which present a different cultural and geopolitical context from Kogi and Ebonyi states [16, 20]. Additionally, our review of numerous studies of care seeking in Nigeria found that the majority focused on fever and malaria [12, 21, 22] and did not examine care seeking across the other, common child illnesses such as pneumonia and diarrhea. The majority of the existing studies were quantitative [16, 18, 23] and did not provide adequate depth into the multiple factors that influence sick-child care seeking, including complex gender and social norms. Our study examines context-specific barriers and facilitators to seeking sick-child care outside the home in Nigeria’s Kogi and Ebonyi states, and contributes detailed information that will support the adaptation of program strategies with a particular emphasis on gender-sensitive approaches for community engagement, and demand creation.

## Methods

### Study design and sample

We conducted a formative qualitative study to explore factors that may enable or inhibit parents from seeking care outside the home for a sick child less than 5 years of age. The study took place in Kogi and Ebonyi states in the four local government areas (LGAs, or districts)—Izzi and Ohaozara in Ebonyi State and Idah and Okehi in Kogi State—where MCSP-supported an intervention to strengthen the quality and access to services delivered at non-clinical private sector Patent and Proprietary Medicine Vendor (PPMV) outlets known colloquially as chemists. We focused on sick children with symptoms consistent with the three leading causes of post-natal child mortality in Nigeria. The study did not seek to explore care seeking based on the biomedical disease classification or caregiver’s ability to distinguish the symptoms of biomedical disease conditions. The purpose of the study was to explain using a grounded theory approach how and why care-seeking behaviors did or did not occur to inform MCSP’s child health programming in these states. The study employed in-depth interviews with mothers and fathers of sick children between 0 and 59 months of age, interviews with in-charges of public health facilities and PPMVs at the community level, and focus group discussions with community leaders such as village chiefs and community health workers. MCSP developed interview guides for the purposes of this study and oversaw pilot testing of the guides by Health Systems Consult Limited (HSCL) interviewers in communities outside Abuja (Supplemental file 1: Interview Guides). Interviews took place face to face in a private location, in the homes of parents, at the place of service for public providers and PPMVs and at community leader meeting points. Participants interviewed reflected the demographic characteristics of the study LGAs. Kogi state LGAs have an equal distribution of Muslim and Christian households compared to Ebonyi state LGAs were nearly all households are Christian. The economy is largely agriculture based and most households have a mobile phone [24]. Mothers in Ohaozara LGA, Ebonyi are more educated than in the other three LGAs with nearly three quarters of women having a secondary or higher education, compared with the average across all LGAs of just over half [25]. Primary health care is available through health centers which provide some medicines for free. But, a large percent of the population seeks care in the private sector and health insurance is largely unavailable to those working outside the formal sector. The four LGAs were selected out of 12 where MCSP was conducting child health activities, using the following criteria: 1) density of PPMV outlets compared to the population size; 2) number of hard to reach communities; and 3) accessibility for MCSP program management. Two LGAs, Onicha and Ikwo, in Ebonyi were excluded due to previous work with PPMVs by MalariaCare. The study sample was designed to enable comparisons across illnesses and LGAs among families who sought and did not seek care. Recognizing that household decision making around care-seeking is a complex process, we interviewed both mothers and fathers to ensure diverse perspectives were represented. To achieve saturation, we proposed to interview a total of 12 mothers (six who sought care and six who did not for the last reported child illness); 12 fathers (six who sought care and six who did not); six primary health facility in-charges and six PPMVs; and three focus group discussions with eight to ten community leaders in each LGA.

### Procedures

Data collection took place from February through March 2018. HSCL, a Nigerian research firm, conducted interviews with participants from the four selected LGAs across the two states with MCSP oversight. The MCSP-led study team interviewed mothers and fathers in the same household who reported having sought or not sought care outside the home for each of the three illness types (fever, diarrhea, or cough). HSCL selected interviewers based on their familiarity with the participants cultural context. Interviewers administered the in-depth interview guide in the participant’s preferred language—largely English, Igala, Igbo, or Ebira after describing the objectives of the research. Participants were selected if they had a sick child under the age of five two weeks prior to a screening questionnaire introduced through a complementary quantitative household survey during which they provided consent to be re-contacted for follow-up data collection in January 2018. The MCSP-led study team used convenience sampling in each LGA to recruit health providers in the same communities of the interviewed parents and purposively selected community leaders for focus group discussions from two communities in each LGA where parents were interviewed. The interviews and focus groups aimed to assess actors and factors that prevent or enable care seeking as described in Colvin et al.’s conceptual framework. We used Colvin et al.’s framework because it captures salient themes from a wide range of qualitative research across sub-Saharan Africa. Male and female interviewers trained in qualitative methods conducted interviews and focus groups in the local languages after obtaining informed consent from participants. Interviews lasted approximately 1 h, and interviewers took notes throughout the process. Interviews were recorded using a digital audio recorder and translated and transcribed into English by the Nigerian research firm HSCL. MCSP staff reviewed a subset of audio recordings to ensure the accuracy of translated transcripts. Due to challenges with the recording devices in Idah LGA, 14 interviews were repeated.

### Analysis

The study team developed an initial set of thematic codes prior to reviewing the transcripts to capture the broad categories reflected in Colvin et al.’s conceptual framework as described in Fig. 1. We then used open coding when reading transcripts to classify emerging themes related to barriers and facilitating factors and gender roles. Three staff from HSCL and MCSP applied codes to the translated English transcripts using NVivo 12 and found agreement across basic themes. Inconsistencies in how codes were applied were resolved after discussion with the principal investigator. We compared findings across states, different illnesses, type of participant, and those who sought biomedical care versus those who visited a chemist, administered self-treatment, or visited a traditional healer. We applied the consolidated criteria for reporting qualitative research (COREQ) checklist to ensure complete and transparent reporting (Supplemental file 2: COREQ checklist).

The ethics committees in the Director of Medical Services offices in the Kogi and Ebonyi state Ministries of Health provided approval for the study and consent forms. The study also received approval from the John Snow, Inc. Institutional Review Board in the United States.

## Results

**Fig. 2:**
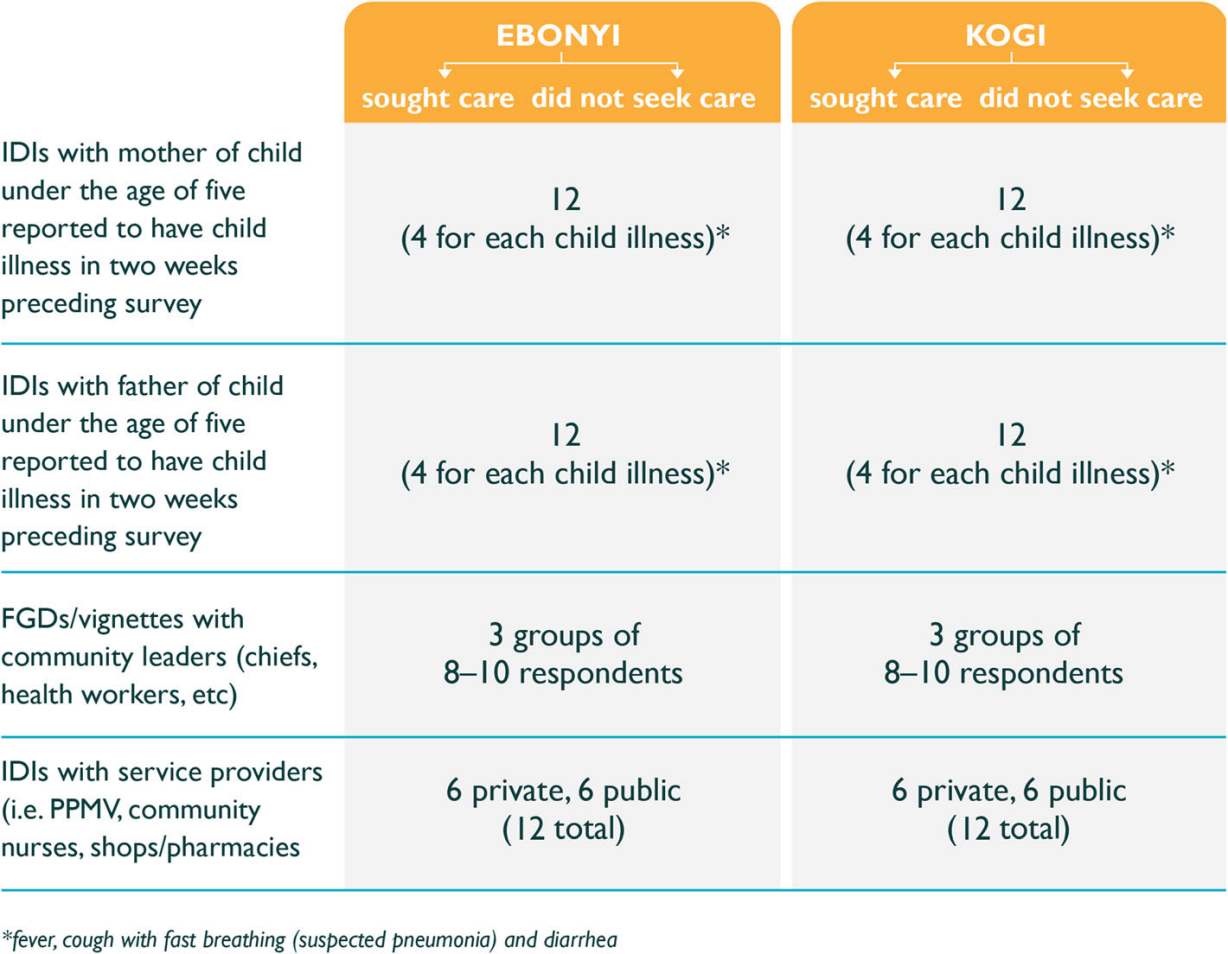
Description of final sample size

We interviewed a total of 12 mothers (six who sought care and six who did not); 12 fathers (six who sought care and six who did not); three primary health center in-charges and three PPMVs; and held two focus group discussions with eight to ten community leaders in each LGA (see Fig. 2). Due to time constraints, the final sample of public and private service providers was reduced from the originally proposed six to three and the focus group discussions among community leaders were reduced from three to two per LGA. We present findings based on the four modes identified in the Colvin et al. framework that influence care-seeking behavior in response to child diarrhea, pneumonia, and malaria.

### Caregiver’s recognition and response to the illness

According to all participants, mothers were the primary caregivers for children, responsible for daily feeding and caring for the child. Interviewers asked parents to describe the symptoms their child had during their most recent illness. Responses from parents were largely consistent across LGAs and care-seeking patterns. In cases where children had diarrhea, parents described loose and frequent stools. For children with a fever, parents described the hotness of their body. Parents who suspected their child had symptoms of pneumonia described symptoms that included coughing, difficulty breathing, and shortness of breath. While these were the primary disease-specific symptoms, parents also described several symptoms that were common across illnesses including, fatigue, weakness, and an inability to eat well or play.

*“I know how that child plays and whenever the child is not able to move around in such manner, I will know that there is a problem somewhere. Aaah when I first returned from the farm that evening, the child was sleeping and I woke the child up in order to feed the child because I know the child would have been hungry by that time, because I stayed long in the farm that very day. (coughing) the child woke up and ate just a little.” (mother, Izzi)*

Many symptoms overlapped across illnesses. For example, children with diarrhea or a cough also had fever. Parents described difficulty breathing, convulsions, extreme weakness, excessively crying, not sleeping, and loss of consciousness as symptoms that indicated the illness was severe. Parents were more likely to seek immediate care if the illness was perceived to be severe. However, all parents reported providing some form of care, regardless of severity, and whether it was first initiated inside the home or not (i.e., traditional, self-treatment, or at a chemist or biomedical provider). Most parents recognized the connection between poor sanitation and hygienic practices and diarrhea. They also mentioned that poor nutrition and teething could contribute to diarrheal illnesses. Many parents recognized the connection between mosquitos and malaria or fever, but sometimes considered the symptom of fever and the medical diagnosis of malaria as the same. A number of parents believed that spending too much time in the sun could cause fever. Nearly all parents and a number of primary health center in-charges interviewed believed pneumonia was caused by being cold or not dressed warmly. Some mothers in Idah LGA attributed pneumonia-like symptoms to certain foods, such as groundnuts.

*“It should be the groundnut that I the mother eat and she takes through the breast milk. The elder sister also gave her groundnut to eat, and it causes cough.” (mother, Idah)*

### Seeking advice and negotiating access to care with the family

Mothers are in close contact with the child and as a result are able to recognize when the child is not feeling well. When the child is ill, mothers typically initiate a conversation about the child with the father. These conversations frequently occur in the evening, when both parents are at home, and can result in a delay in seeking care if the illness began earlier in the day. The discussions center on the child’s symptoms and severity, the type of treatment that is needed, whether money or transport is required for care seeking and the quality of the provider. Mothers who have access to a cell phone may choose to reach out to the fathers earlier in the day. In general, mothers and fathers reported finding agreement in their care-seeking decisions. The discussions typically remain within the nuclear family, between the mother and father unless the case is particularly serious and requires resources beyond their means.

*“I had a discussion with my wife that night, when the coughing started, like I said we could not go out because it was late, so she suggested we wait till morning before we could take the child out to seek care in the hospital in Okene. Before the morning she came to me again that we would have to leave early in the morning because the cough had not stopped and it looked like it was getting worse.” (father, Okehi)*

Strong gender norms relating to childcare and decision-making emerged during the interviews. The social and gender norm themes observed were consistent from both the mothers and father’s perspective. Across all four LGAs, participants agreed that the role of the father is to financially support the child and provide money for treatment.

*“The mother takes care of the sick child; the father’s own is to bring the money” (mother, Okehi)*

The mother is responsible for providing physical care for the child and taking the child for treatment. Fathers will take the child for treatment if the mother is not available or will occasionally accompany the mother to the biomedical provider, particularly if the illness is perceived to be severe or if he has access to a means of transportation. Because the father is usually responsible for paying for the care, he ultimately makes the decision about when and where the child receives care. A number of participants acknowledged that a mother would delay care seeking if money was not available from the father and that the child’s illness could become increasingly severe as a result. Deviations from this norm were only apparent when the mother has financial autonomy. In this case, both mothers and fathers agreed that a woman could contribute resources for care and in some situations take decisions without permission from the husband.

*“What other duty do we have if not to make sure that the children are healthy. The man will make sure that money for the treatment is provided but if a woman has she would bring too. It’s not like it must be the man” (mother Ohaozara).*

In addition to financial support, participants from Kogi and to a lesser extent in Ebonyi indicated that cultural norms dictate that the child belongs to the father and the mother does not have the right to seek care without permission from the father. If the mother takes the child without permission from the husband, he could refuse to pay the bill or send her back to her father for disobeying him, or hold her responsible for the child’s illness or death. The belief that a child belongs to a father independent of a woman’s financial autonomy was more common in Kogi state, perhaps due to the higher percentage of Muslim communities.

*“she needs permission from me or my father. This is because if anything goes wrong she will be blamed for not telling anybody.” (father, Ohaozara)*

In some situations when the father is away, family members such as a brother of the father may be assigned to help the wife if there is a problem. The relative may provide financial support or advice, particularly if they have more experience with children.

Most parents acknowledged that aside from limited prevention efforts promoted at the community level, the community members did not play a significant role in advising on or supporting care seeking and that these decisions primarily occurred in the home. Some caregivers did note that community members provided prayers and advice, checked in on the family, collected herbs to support treatment, and provided financial or logistical support if the family did not have the resources to obtain care. In Idah LGA in Kogi state, a number of participants spoke about the social cohesion within the community. Some mothers in Idah LGA said that community leaders could provide permission for the mother to seek care outside the home if the father was not available. All participants noted that community members did not prevent parents from seeking care for their child.

### Making use of community based treatment options (i.e. the “middle layer” between home and clinic)

Even if parents did not seek care outside the home, parents initiated some form of care using herbal remedies or medicines available at home.. A number of parents mentioned that traditional remedies are an effective means of treating a sick child, and this is based on experiences passed down through families.

*“How I know about the drugs is that we do follow our father and go inside the bush and our father will get leaves and administer on a sick person and the person will be well” (father, Ohaozara)*

Mothers and fathers learn from their parents which herbs are appropriate for different illnesses. Some mothers noted that certain herbs can help clean the stomach from the illness or that other traditional remedies such as palm kernel oil can help release the heat from the body during a fever episode. A mother may apply these remedies at night to soothe a child before they seek care in the morning. Traditional remedies are often seen as a more cost-efficient form of treatment since they are available freely in the bush. The only disadvantage seen in using herbs is that they can be time consuming to prepare.

Participants from Idah LGA in Kogi spoke of traditional healers who diagnose and advise on how to treat children. Idah community leaders mentioned that the traditional herbs used to treat the children are passed down through the community elders and are similar in efficacy to those found in the hospital, and that the hospitals have access to more herbal medicines than those found in the community.

However, communities in Ohoazara LGA in Ebonyi believe that times have changed and parents rely less on traditional approaches.

*“Their duty is when the parents notice that a child is sick, you ask for their advice from elderly people. In those days, they use kola, chicken blood, etc., to do some ritual and then tell you that the fever has been cured and it actually does. It is the grandfather and grand grandmothers that guide us then. Now it is no longer like that.” (male community leader, Ohaozara)*

At times, particularly if the mother did not perceive the illness as severe, she would try to treat the child at home based on her knowledge from previous experiences with a biomedical provider or with medications leftover from a previous illness or purchased from a local chemist. For example, a number of mothers mentioned that they kept paracetamol at home and administered it to the child if they had a fever or applied balms and gave the child a hot bath to alleviate congestion. However, if the medications did not improve the condition, the mothers would seek another form of care.

In Nigeria, chemists or PPMVs are frequently the first source of care for under-five child illnesses. In addition to selling drugs, PPMVs can be a source of advice about illness and drug therapy in place of more formal care at health facilities even though most PPMVs have not received formal training in prescribing pharmaceuticals [21]. Parents perceive chemists to provide good-quality care, but that they may not be equipped to handle serious illnesses. Some parents will frequent a chemist after receiving a prescription from a biomedical provider, while others will seek advice directly from the chemist on the type of medication to purchase. The families’ proximity to the chemist compared to the distance to a health facility also influences their choice, particularly if they are perceived to have drugs available of good quality and at a good price.

*“They only way we don’t provide quality care is maybe they are not with enough money, so we do compromise. We give them on credit and they later come to pay. Unless if the person doesn’t want to buy on credit, we give them alternatives, or the person would just leave because he cannot afford the bill.” (male, PPMV, Idah)*

Some families have a relationship with the chemist. The mother can take the child to the chemist when the father is away and the chemist can provide services on credit with the agreement that the family will make the payment in a few weeks.

***“****It is the one that I can afford. I used to go to the hospital but my husband was not around to give me money to give them. The chemist use to give me medicine, and when my husband comes back, he pays for it.” (mother, Ohaozara)*

### Accessing formal biomedical services

Decisions on when and where to seek care were multidimensional and did not always follow a linear path. Care seeking moved between providers, with some parents seeking care at chemists and then continuing to health facilities or starting with a health facility and then accessing prescriptions from a chemist. While some participants have maintained their trust in traditional medicines, others noted Christian religion encourages Western medicine. Depending on factors such as illness severity, financial resources, and illness duration, some families will try traditional remedies first, and if the illness continues or becomes more severe, they then seek care with a chemist or biomedical provider at the health center or hospital. The sex of the child does not influence decisions on whether or not to seek care or where care is sought, according to all participants asked.

*“We have been used to herbal medicine and occasionally we subscribe to orthodox medications. The other day, my child took ill; after several herbal medications without improvement we went to the clinic and somehow, he was greatly relieved. However, we will be glad if we can have the orthodox medication cheaply, we will prefer it.” (mother, Idah)*

Referrals occurred when a mother takes the child to a chemist, but the chemist is unable to offer the appropriate treatment.

Parents were hesitant to acknowledge cost and distance as a barrier to care seeking and emphasized the importance of the child’s life, whereas providers and community leaders perceived these issues as significant barriers for families. However, some families in Idah LGA in Kogi state acknowledged that poverty meant they did not have money to seek formal treatment and they were forced to resort to herbal remedies to treat child illnesses.

*“Money can actually delay the mother from seeking care; as a mother when you don’t have money, you would have to wait for the father. Money is a great factor. Imagine you get a prescription from the nurse, on getting to the chemist without money, nothing can be done.” (male community leader, Idah)*

Parents and public and private health care providers acknowledged that families could access treatment on credit. Both PPMVs and biomedical providers said they would not refuse to provide care to a sick child if the family did not have money and they would search for the means to treat the child.

Community leaders perceived biomedical providers to be inaccessible to the poor because even though services are provided free of charge, transportation to the facility, obtaining laboratory tests and treatment are considered expensive. However, when an illness is severe or the child is not improving, mothers will seek treatment at a health facility.

*“I considered it to be severe when the child did not respond to the drugs I have. I now knew that the case was beyond chemist, I then took her to the hospital. The child was coughing, and the body was hot.” (mother, Ohaozara)*

Parents perceive a number of advantages when visiting the health facility. First, providers are more likely to administer a diagnostic test, which is considered a higher level of care because the provider is confirming the illness through a test and not simply providing care based on symptoms. Parents also expect short waiting times and strong interpersonal skills from providers across all levels of formal care. While parents consider these examples of good quality care, the ultimate measure of quality is that the child recovers.

## Discussion

This study examines the complex dynamics surrounding decisions to seek care for sick children in the North Central and South West zones of Nigeria. Our findings organized by the Colvin et al. (2013) framework [13] informed community engagement strategies and messages around appropriate care seeking for sick children through MCSP’s child health program in Nigeria. We identify several context-specific barriers and facilitators to seeking sick-child care outside the home that can inform the development and adaptation of future program strategies with an emphasis on gender-sensitive approaches for community engagement, and demand creation.

### Barriers to seeking care for sick children outside the home

We found that while both parents had a general understanding of illness symptoms, families and communities attributed illnesses to both biomedical and naturalistic causes, which can hamper prevention and treatment. Some mothers perceive illnesses such as fever and diarrhea to be caused by teething, which is a normal part of early childhood and may not prompt early care seeking, as seen in previous studies [26, 27]. We also found that some participants do not have a complete understanding of the symptoms of pneumonia and attributed pneumonia symptoms to weather, being cold or certain foods, similar to findings from other areas in Nigeria and elsewhere in sub-Saharan Africa [28, 29]. Program should consider engaging with community volunteers or through other communication campaigns to build awareness on the causes of childhood illnesses.

We found that gender, and sociocultural roles and norms within the home play an important role in care seeking for child illnesses in Kogi and Ebonyi states, Nigeria. Our findings add to a growing body of evidence that suggests that public health programs that exclusively target mothers of young children risk failure when they do not take into account the complexity of intra-household dynamics [8, 19, 30, 31]. Seeking permission from a male partner can be a delay in seeking timely care for sick children. Addressing this delay in care-seeking requires gender-sensitive programming that engages men and community leaders in health education and promotes equitable decision-making [24, 25]. Community dialogues with small groups of male leaders have been found to motivate fathers to act as change agents and enable them to encourage other community members to assist with maternal health needs in the community in Nigeria [32]; these could be expanded to include child health interventions [33]. Programs can also introduce a community based non-monetary incentive to support male attendance at community information sessions to strengthen their understanding of their role in supporting mothers in recognizing and seeking care for sick children [34]. Given the role and status of mothers as primary caregivers, future studies should consider to what extent they are instructed by others on what to do; how often they understand and are in agreement with these instructions; how they circumvent instances when delays occur, when fathers are unavailable, or when inappropriate decisions are directed; and what factors enable them to take appropriate action in these situations [35]. Strengthening men’s involvement and community support to enhance mothers’ agency, linking to community-based options, and ensuring access to biomedical care should also be subjects for future programming and study.

Previous studies have found that cultural beliefs can play an important role in seeking care for child illnesses; that families often use a combination of biomedical and herbal treatments; and that traditional healers and treatments are commonly available in the community and often used during the onset of illness [14, 36, 37]. They also indicate that parents trust traditional healers and medicines to treat illness and that this contributes to delays in care seeking from biomedically oriented providers [38, 39]. Our study similarly found use of traditional medicine, as well as self-treatment, to be a common first step in sick-child care seeking. Before accessing biomedical services, self-treatment, particularly using leftover medicines from a previous illness or buying medicines previously prescribed for a similar illness, was a common first step among parents. This finding aligns with previous quantitative studies in Lagos that found that many caregivers purchase previously prescribed medicines or rely on medicines leftover in the home to treat the child [35]. While home-based treatment is a common first step for families, the diversity, adequacy and sequencing of home care is not well understood [40, 41]. Future studies should consider developing a standardized typology for the type of treatment provided that would enable comparisons across different contexts [42].

We found that providers and community leaders identified financial barriers to care seeking. However, most parents said that financial and geographic barriers did not influence care seeking. Previous studies have found that the ability to access biomedical services, both financially and in terms of the distance to the service, as well as the quality of services previously received, also has a significant influence on care seeking for child illnesses [12, 14, 15, 21, 22, 43]. However, Kamat et al. argue that overemphasizing financial barriers may distract from the cultural barriers that delay care seeking [44], such as intra-household decision-making norms or preferences for traditional medicine or self-treatment.

### Enabling factors to seeking care for sick children outside the home

Our findings suggest that parents in Ebonyi and Kogi states recognize severe illness symptoms that should prompt immediate care seeking, in contrast to findings from elsewhere in Nigeria [30].

Similar to other studies, we found that mothers are most frequently the first ones to identify illness symptoms [23], but that a mother often does not have the agency or resources to seek care herself and must consult her husband or in-laws prior to seeking care outside the home [16] and decisions about where and when to seek care for a child are often taken by fathers, since men are considered to be responsible for family resources and pay for care [8, 19, 30]. Despite these limitations on a woman’s agency, increased access to mobile phones have made it easier for mothers to contact and obtain a father’s permission while he is away. Health program managers should build on the local knowledge of severe symptoms and develop health education campaigns targeted to families and health care providers to increase knowledge of the causes of child illnesses and reinforce prompt care seeking for severe illness, especially for pneumonia. Encouraging spousal communication though mobile phones about care seeking may also help to ensure that couples are able to make more timely decisions about care that improve child health outcomes.

We identified some variation across LGAs with regard to making use of community based treatment options. For example, some participants in Ohaozara LGA in Ebonyi felt that cultural traditions related to care seeking were changing in favor of Western medicine, while participants in Idah LGA in Kogi state appeared to retain more of the traditional values, including a degree of social cohesion and community support that could, in the case of a father’s absence, enable community leaders to provide a mother permission to seek care for a sick child. The social ties in more traditional communities could be leveraged to increase a mother’s access to advice on appropriate treatment or to provide financial support. Engaging community members as active participants in supporting a mother can help to shift social norms towards positive health behaviors and create social support that improves health outcomes [34]. Program managers should consider adopting a community readiness model to better understand how communities can be engaged to overcome cultural and gender norms that may serve as a barrier to seeking biomedical care for sick children [45].

When parents sought care outside the home, they often went to PPMVs due to their geographic proximity and because they perceived quality medications were available at an affordable price. MCSP worked in LGAs to improve the quality of care and medicines at PPMV outlets, as well as to strengthen referral of children with danger signs to a higher level health center. As part of the MCSP program, community-based organizations encouraged parents to seek care at trained PPMVs/chemists for simple childhood illnesses and to seek care at health centers for more severe symptoms. Program managers and policy makers should continue to explore opportunities to strengthen the functioning of primary health centers in Nigeria and to leverage the role of chemists in making referrals and supplying quality medicines [46].

### Limitations

While previous studies acknowledge that recognition and response to childhood illnesses involves household and community members beyond the primary caregiver which is usually the mother, our study did not interview the extended family or friends (i.e. mothers in law, grandmothers, aunts, etc) in order to limit the scope of the study. Socio-demographic characteristics of study participants (including the sex of community leaders and health providers) were not systematically captured during data collection and therefore could not be used during analysis.

## Conclusion

Behavior change communication related to child health focuses primarily on the mother who is the primary caregivers responsible for symptom recognition and taking children to the health center for care. Our study identified barriers and enabling factors within the household, at the community level and in accessing formal biomedical providers that suggests programs should more actively engage actors at various levels to strengthen recognition and timely response of care for sick children. While fathers take financial responsibility for a child’s treatment, strong opinions on the father’s role in determining if or when a child receives care can result in delays in care seeking, particularly when the mother is the primary caregiver of the child. Community members can play a supportive role in empowering a mother to seek care when a father is unavailable. Future programs should consider the household roles and intra-household dynamics when planning interventions and seek to foster an environment where mothers are empowered to make decisions related to care seeking for sick children.

## Supplementary information

**Additional file 1: Supplemental file 1.** Interview Guides.

**Additional file 2: Supplemental file 2.** COREQ checklist.

## Data Availability

All data relevant to this publication can be obtained by request to the authors.

## Abbreviations

MCSP: Maternal and Child Survival Program
LGA: Local government authority
PPMV: Patent and Proprietary Medicine Vendors
