# Supplementary material for: Understanding factors influencing care seeking for sick children in Ebonyi and Kogi States, Nigeria
Source: BMC Public Health. 2020 May 24;20:746. doi: 10.1186/s12889-020-08536-5 (PMC7245913; doi:10.1186/s12889-020-08536-5)
Supplement: Supplementary file 1 — Additional file 1: Supplemental file 1. Interview Guides. [file 12889_2020_8536_MOESM1_ESM.docx]

**Combined Interview Questions for the Qualitative Research: Understanding barriers and facilitating factors in seeking care for sick children under the age of 5 in Ebonyi and Kogi states, Nigeria.**

Instructions to interviewer and/note taker: To begin the interview, introduce yourselves and the project. Below is a suggested introduction. It does not need to be followed word for word, but key messages should be conveyed. Interviews should be recorded and notes should be written down in a notebook and later expanded and typed up, adding in the date recorded in the header below (e.g. interviewer name, date). Pose questions in a neutral manner, listen attentively to participants’ responses, and ask follow up questions and probe for as much information/explanation/description that is relevant to the questions as possible. Be sure to familiarize yourself with the interview guide prior to initiating the discussion, and to thoroughly understand the overall objectives of this research—you may be called on to rephrase questions that are unclear to participants, or to spontaneously think of follow-up questions or probes. Avoid leading or influencing participant responses based on preconceived notions or encouraging them to provide particular answers. Remember that we will use these data to understand respondents’ perceptions of their experience and not simply describe the objective “truth” of their experience.

The interview should last about 90 minutes.

Interview identifier

| State |  |
| --- | --- |
| LGA |  |
| Community |  |
| Interviewer |  |
| Note taker |  |
| Interview Date |  |
| Start time |  |
| End Time |  |

Interviewers: Thank you for taking the time to meet with us. My name is ____________

Thank you for agreeing to this visit and interview. The Maternal and Child Survival Program (MCSP) is working with the State Primary Health Care Development Agency (SPHCDA) to improve child health through providing better services. A few weeks ago, our colleagues were here to ask you some questions about some diseases that occur in children under 5 years. We will also be asking similar questions, but our questions will be focused on understanding how you care for your children aged 0-5 years when they have either malaria, diarrhea or pneumonia.

Would you be willing to provide valuable feedback on your recent experiences caring for a sick child?

We are grateful that you are willing to take the time to speak with us.

Throughout this interview, ***I will be asking some questions about your opinions of and experiences with caring for a sick child.*** We will take notes and record our conversation.

**CONSENT SCRIPT FOR VERBAL CONSENT**

Your participation in this interview is completely voluntary. The interview will not take more than about 90 minutes, and there is no right or wrong answer to any of the questions.

***If you choose to participate, there is a risk that you may share some personal information by chance, or that you may feel uncomfortable talking about some of the topics.*** We do not wish for this to happen. You may withdraw your consent and discontinue participation at any time and you have the right to refuse to answer any or all questions. There will be no direct benefit to you for participating, but ***your participation may help us learn about ways to improve the health of mothers and children. We will not be sharing information about you to anyone outside of the research team and the personal information that we collect from this research will be kept private and anonymous.*** Any information about you will have a number on it instead of your name. When this study is completed, a summary report of our research findings will be written up.

Do you have any questions?

***Do you give your verbal consent to participate in this research***? Yes___ No___

I want to thank you in advance for your participation in this study.

Prior to starting the interview, confirm each participant answers yes to the screening questions below.

1. Have you had a child in the last five years? ___Yes ____No

When our colleagues were here, you told them that one of your children had just recovered from pneumonia/ malaria/ diarrhea. (Choose the correct information as appropriate – just the disease as specified in your respondent information sheet)

1. Was your child sick with diarrhea, pneumonia or fever? ___diarrhea ____fever ____pneumonia
2. Did you seek treatment for the child? ___Yes ____No
3. Introduction of Respondent (Warm- Up): Please tell me a little bit about yourself.

| Respondent Type |  |
| --- | --- |
| Respondent Code |  |
| Age |  |
| Level of Education |  |
| Occupation |  |
| Number of Children |  |

**PARENTS WHO SOUGHT CARE**

| *Objectives* | *Themes* | *Questions* |
| --- | --- | --- |
| **Objective 1: Explore how presentation, recognition and interpretation of illness signs and symptoms in the household influence care seeking for sick children.** | *Presentation and Recognition of illness signs and symptoms* | *- Can you tell me how you knew your child was sick or how the illness started? (Probe for time of the day details of what the respondent and the child were doing when the illness was first noticed)*  *-What were some of the signs you first noticed?* |
|  | *Severity* | *-* *Which of these signs in your child concerned you the most?*  *-How did the signs or illness change during the first day and then from the first day to the second day? From the second to the third day?*  *-Was the child very ill, and if so how could you tell that the child was very ill? (Probe for specific signs, behaviours or other circumstances)* |
|  | *Causes* | *-* *What do you think caused the illness?* |
|  | *Treatment* | *-* *What did you do about the child’s signs or illness?*  *-Why did you decide to take this action? (Probe for recognition of the signs and symptoms, severity, presence/absence of a particular symptom, knowledge of causes, proximity to healthcare provider)*  *-Did you seek care outside the house or did you call for someone to come to the house to take care of the child/illness?*  *-Where did you seek treatment? Why did you choose this source? Did you take the child with you when seeking treatment?*  *-* *What treatment was given to the child*? *Who gave this treatment? Where did (s)he or you give these treatments to your child? How did (s)he or you give this treatment to the child? How often? Did (s)he or you have any trouble providing the treatment?*  *-What did you like about this treatment? Will this make you choose this treatment again? What did you not like about the treatment? Will this make you not use this treatment again?* |
|  | *Timing of care* | *- How long did you wait after the onset of the signs or illness before you sought care for the child?* |
|  | *Understanding of treatment for diarrhea* | *-Have you ever heard of “dehydration”? What is it? Do you think your child experienced it? What can be done about it? (explain what dehydration is – e.g. child’s urine was very little and/very yellow, sudden and excessive weight loss, when you press a part of the child’s skin, it doesn’t return to normal immediately, sunken fontanelles, the child looks shrunken like an old person, when the child cries no tears come out of his eyes)*  *-When the child had diarrhoea, did you give more or less to drink than usual?*  *-What specific fluids did you give your child when (s)he had diarrhea? Why did you decide to provide fluids?*  *-Tell me about how you prepared fluids for your child. (probe for kind of bottle or container it was prepared in, kind/ source of water (fluid) used, how many times daily it was given, how much was given, how many days after preparation it was given to the child)*  *-How did the child respond to the fluids?*  *-Has someone ever demonstrated to you how and how much fluids to provide to your child when it had diarrhoea? Who and where?*  *-When your child had diarrhoea, did you feed your child differently? How so? How did the child respond to the food?*  *-Tell me about the treatment you gave to the child:*  *-What medicine did you give to the child? why?*  *-Who gave you this medicine? Did the person explain to you how much and when to give this medicine? Did you have any problems to give the medicine as told?*  *- (Mothers only) Are you currently, or before the child became sick, breastfeeding the child? Did you continue breastfeeding when your child had diarrhoea? Did you breastfeed more or less?* |
|  | *Understanding of treatment for fever* | -*Have you ever heard of “malaria”? What is it?*  *-how do you know when a child has malaria? Do you think that was the illness your child had? How do you differentiate between malaria and fever? What can be done about it?*  *-Tell me about the things you did when your child had fever (probe for specific medicine, home care or remedies used) Why did you use/ do these things?*  *Let’s talk about the medicine you gave the child*  *Who gave you this medicine? Did (s)he explain to you how much and when to give this medicine? Did you have any problems to give the medicine as told?*  *-Did someone show you how and how much to give to your child when (s)he had malaria? Who and where? Was there a separate medicine for the different signs of malaria?*  *-How did the child respond to the medicines or remedies?*  *- When your child had malaria, did you feed your child differently? How so? How did the child respond to the food?*  *- (Mothers only) Are you currently breastfeeding? Did you continue breastfeeding your child once the malaria started?* |
|  | *Understanding of treatment for pneumonia* | -*Have you ever heard of “pneumonia”? What is it? What are the signs? Do you think your child experienced it? What can be done about it?*  *-Tell me about the things you did when your child had pneumonia (probe for specific medicine, home care or remedies used) Why did you use/ do these things?*  *-Let’s talk about the medicine you gave the child*  *Who gave this medicine? Did (s)he explain to you how much and when to give this medicine/remedy? Did you have any problems to give the medicine as told?*  *-Did someone show you how and how much to give to your child when (s)he had pneumonia? Who and where? Was there a separate medicine care or remedy for the different signs of pneumonia?*  *-How did the child respond to the medicines or remedies?*  *- When your child had pneumonia, did you feed your child differently? How so? How did the child respond to the food?*  *- (Mothers only) Are you currently breastfeeding? Did you continue breastfeeding your child once the malaria started?* |
| **Objective 2: Understand how women, men and family members interact in terms of seeking advice and negotiating access to care.** | *Household gender roles related to childcare* | *- This last time your child was sick, who took the child to seek for care?*  *-Do you consider it your role to seek care for the child when ill?*  *(probe for reasons why the care giver does not consider it his/her role e.g. culture, gender roles, finances, religion)*  *-Is it also anyone else’s role in the family? (probe by gender – grandmother vs grandfather, sister vs brother…)* |
|  |  |  |
|  | *Decision-making conversations* | *-* *Did your family provide advice regarding how to treat or take care of your child when s/he was sick? Did your family advice you about where you sought care?*  *-IF YES, Probe for*  *-Who gave advice; (father/ mother of the child, grandmother, other family members, non-family members)*  *-Who initiated the conversation;*  *-When the conversation occurred (i.e. how soon after the illness began)?*  *Where the conversation took place*  *-What the discussion was about? (probe for cost of treatment, proximity diagnosis, severity)*  *Did you follow their advice? (father of the child, grandmother)*  *- What did you think about the advice you received? How did it make you feel?*  *-Did you get any advice that seemed wrong or that you did not agree with?*  *-Did anyone in your family discourage you from going to seek treatment? If so, can you tell me more about what they said and why?*  *- What type of support did you receive when the child was sick?*  *PROBE QUESTIONS:*  *-Did you live with the father/ mother of the child when the child was sick?*  *-How Did the father/ mother of the child support you to seek care for your child?*  *Tell me some of the specific things the father/ mother of the child did, if anything.*  *-How did your mother-in-law support you? (tell me some of the things your mother in law did.)*  *-If there were any costs for the transport or the health services, who paid for them?*  *-Did the father/ mother of the child accompany you when seeking care?*  *- Did other family members or non-family members provide you with any support?*  *-What other types of support have you received? How did it make you feel?* |
|  | *Negotiating Access to care* | *-* *Do you require permission to seek care for your sick child outside the home? If so, from who?*  *-What factors determine who decides to seek care for an ill child (probe for effect of customs, gender, head of household, finances etc.)* |
| **Objective 3: Explore how social norms, gender roles, and financial and geographic access influence treatment and care seeking decisions.** |  |  |
|  | *Location* | *-* *How did you reach the treatment place? How long did it take to get there? Once there, how long (in minutes) did you have to wait to receive a consultation?*  *-Does the time it takes to get there or distance of the place from your home determine where you take your child for treatment when (s)he is sick? If yes, why? If no, why not?*  *-Does transport fare determine what treatment and WHERE you seek care for a sick child?* |
|  | *Finances/ Out of Pocket Expenses* | *-* *How much money did you spend on treatment (probe for amount of money spent where treatment was received, amount of money spent on the home remedies used, new foods or drinks introduced because of the illness, other in-kind payments that had to be made relating to making the child better)*  *- So, if you add all these things together, do you think that is a good price?*  *-Does cost of services determine WHERE you seek care for a sick child? How about the ease of paying (e.g. buying on credit, paying small-small) for services determine WHERE you seek care for a sick child?*  *-Does cost determine WHEN you seek care for a child?*  *- Do you consider your role to pay for the care”? (probe for reasons why the care giver does not consider it his/her role e.g. culture, gender roles, finances, religion)* |
| **Objective 4: Understand how previous experiences and perceptions of quality of care across types of providers affect care seeking patterns for sick children.** | *Perceptions of Quality of Care (related to the last illness episode)* | *This last time your child was sick, how many health providers did you visit?*  *How reliable/trustworthy did you find each of /the provider(s) you visited in the last illness episode?*  *-Did you have a good or bad experience with this source of care?*  *-Did the illness continue? If yes, what did you do next?*  *-Are you glad that you decided to seek care outside of the home? Why or why not?*  *-Do you think that you were given a proper treatment for your child’s illness?*  *-Would you return to the same place for treatment if your child became sick again? If yes, why? If not, why not?*  *-If the care-giver went to more than 1 health provider during the last illness episode: Which treatment(provider) do you think was the most effective? Which treatment was the least effective?*  -What are the things you will see/ experience that will make you say that the health service is good?  *Probes:*  *is it determined by how helpful the health provider is?*  *is it determined by what happens to the child?*  *is it determined by how long you had to wait before you were attended to?*  *is it determined by how well the health provider was able to answer your questions?*  *is it determined by how the health provider treated you?*  *is it determined by how well the health provider understood and considered specific things you asked for?*  *-Based on all these things, which health provider would you prefer to seek care for your child? (probe for service provider type – traditional healer, PPMV, PHC, Private provider etc.)* |
|  | *Previous experiences with different providers*  *(not related to the last previous illness episode)* | *-Tell me about your experience seeking care for your child/ children. Which providers in this community have you visited?*  *Explore the following questions for each provider:*  *When you went to the provider for treatment, how did the provider respond?*  *Did the provider explain the illness?*  *Did the provider explain treatment?*  *Did the provider listen to your questions/ concerns?*  *Did the provider answer your questions/ concerns?*  *What treatments were provided if any? How did child/ children respond to the treatment?*  *- Did any previous experience (good/ bad) influence where you sought care* |
| *Interviewer: Thank you for your answers in the previous section. Now let us talk about the things that happen in your community about seeking care for a sick child under-5* | | |
| **Objective 3: Explore how social norms, gender roles, and financial and geographic access influence treatment and care seeking decisions.** | *Barriers and facilitating factors related to the communities’ social norms and gender roles* | *- In this community, when a child is sick, how does a mother usually respond? Explain.*  *-How do community/religious leaders influence care seeking for a sick child?* |
| **Objective 2: Understand how women, men and family members interact in terms of seeking advice and negotiating access to care.** | *Social Norms* | *Are there any practices/ beliefs in your community that influences seeking advice for a sick child?*  *Are there any practices/ beliefs in your community that influences how you get access to care outside the house for a sick child?* |

**PARENTS WHO DID NOT SEEK CARE**

| *Objectives* | *Themes* | *Questions* |
| --- | --- | --- |
| **Objective 1: Explore how presentation, recognition and interpretation of illness signs and symptoms in the household influence care seeking for sick children.** | *Presentation and Recognition of illness signs and symptoms* | *- Can you tell me how you knew your child was sick or how the illness started? (Probe for time of the day details of what the respondent and the child were doing when the illness was first noticed)*  *-What were some of the signs you first noticed?* |
|  | *Severity* | *-* *Which of these signs in your child concerned you the most?*  *-How did the signs or illness change during the first day and then from the first day to the second day? From the second to the third day?*  *-Was the child very ill, and if so how could you tell that the child was very ill? (Probe for* specific *signs, behaviours or other circumstances)* |
|  | *Causes* | *-* *What do you think caused the illness?* |
|  | *Treatment* | *-* *What are the things you did about the child’s signs or illness?*  *-Why did you decide to take this action? (Probe for recognition of the signs and symptoms, severity, presence/absence of a particular symptom, knowledge of causes, proximity to healthcare provider)*  *-Did you seek care outside the house or did you call for someone to come to the house to take care of the child/illness?* |
|  | *Timing of care* | *- How long after the onset of the signs or illness was it before you did something about the child’s illness?* |
|  | *Understanding of treatment for diarrhea* | *-When the child had diarrhoea, did you give more or less to drink than usual?*  *-Have you ever heard of “dehydration”? What is it? Do you think your child experienced it? What can be done about it? (explain what dehydration is – e.g. child’s urine was very yellow, sudden and excessive weight loss, when you press a part of the child’s skin, it doesn’t return to normal immediately, the child looks shrunken like an old person, when the child cries no tears come out of his eyes)*  *-What specific fluids did you give your child when (s)he had diarrhea? Why did you decide to provide fluids?*  *-Tell me about how you prepared fluids for your child. (probe for kind of bottle or container it was prepared in, kind/ source of water (fluid) used, how many times daily it was given, how much was given, how many days after preparation it was given to the child)*  *-How did the child respond to the fluids?*  *-Has someone ever demonstrated to you how and how much fluids to provide to your child when it had diarrhoea? Who and where?*  *-When your child had diarrhoea, did you feed your child differently? How so? How did the child respond to the food?*  *-Tell me about the treatment you gave to the child:*  *-What medicine did you give to the child? why?*  *-Who gave you this medicine? Did the person explain to you how much and when to give this medicine? Did you have any problems to give the medicine as told?*  *- (Mothers only) Are you currently, or before the child became sick, breastfeeding the child? Did you continue breastfeeding when your child had diarrhoea? Did you breastfeed more or less?* |
|  | *Understanding of treatment for fever* | -*Have you ever heard of “malaria”? What is it?*  *-how do you know when a child has malaria? Do you think that was the illness your child had? How do you differentiate between malaria and fever? What can be done about it?*  *-Tell me about the things you did when your child had fever (probe for specific medicine, home care or remedies used) Why did you use/ do these things?*  *Let’s talk about the medicine you gave the child*  *Who gave you this medicine? Did (s)he explain to you how much and when to give this medicine? Did you have any problems to give the medicine as told?*  *-Did someone show you how and how much to give to your child when (s)he had malaria? Who and where? Was there a separate medicine for the different signs of malaria?*  *-How did the child respond to the medicines or remedies?*  *- When your child had malaria, did you feed your child differently? How so? How did the child respond to the food?*  *- (Mothers only) Are you currently breastfeeding? Did you continue breastfeeding your child once the malaria started?* |
|  | *Understanding of treatment for pneumonia* | -*Have you ever heard of “pneumonia”? What is it? What are the signs? Do you think your child experienced it? What can be done about it?*  *-Tell me about the things you did when your child had pneumonia (probe for specific medicine, home care or remedies used) Why did you use/ do these things?*  *-Let’s talk about the medicine you gave the child*  *Who gave this medicine? Did (s)he explain to you how much and when to give this medicine/remedy? Did you have any problems to give the medicine as told?*  *-Did someone show you how and how much to give to your child when (s)he had pneumonia? Who and where? Was there a separate medicine care or remedy for the different signs of pneumonia?*  *-How did the child respond to the medicines or remedies?*  *- When your child had pneumonia, did you feed your child differently? How so? How did the child respond to the food?*  *- (Mothers only) Are you currently breastfeeding? Did you continue breastfeeding your child once the malaria started?* |
| **Objective 2: Understand how women, men and family members interact in terms of seeking advice and negotiating access to care.** | *Household gender roles related to childcare* | *-Do you consider it your role to seek care for the child when ill?*  *probe for reasons why the care giver does not consider it his/her role (e.g. culture, gender roles, finances, religion)*  *-Is it also anyone else’s role in the family? (probe by gender – grandmother vs grandfather, sister vs brother…)* |
|  |  |  |
|  | *Decision-making conversations* | *-* *Did your family provide advice regarding how to treat or take care of your child when s/he was sick?*  *-IF YES, Probe for*  *Who gave advice; (father/ mother of the child, grandmother, other family members, non-family members)*  *Who initiated the conversation;*  *When the conversation occurred (i.e. how soon after the illness began)?*  *Where the conversation took place*  *What the discussion was about? (probe for cost of treatment, proximity diagnosis, severity)*  *Did you follow their advice? (father of the child, grandmother)*  *- What did you think about the advice you received? How did it make you feel?*  *-Did you get any advice that seemed wrong or that you did not agree with?*  *-Did anyone in your family discourage you from going to seek treatment? If so, can you tell me more about what they said and why?*  *- What type of support did you receive when the child was sick?*  *PROBE QUESTIONS:*  *-Did you live with the father/ mother of the child when the child was sick?*  *-How Did the father/ mother of the child support you to seek care for your child?*  *Tell me some of the specific things the father/ mother of the child did, if anything.*  *-How did your mother-in-law support you? (tell me some of the things your mother in law did.)*  *- Did other family members or non-family members provide you with any support?*  *-What other types of support have you received? How did it make you feel?* |
|  | *Negotiating Access to care* | *-* *Do you require permission to seek care for your sick child outside the home? If so, from who?*  *-What factors determine who decides to seek care for an ill child (probe for effect of customs, gender, head of household, finances etc.)* |
| **Objective 3: Explore how social norms, gender roles, and financial and geographic access influence treatment and care seeking decisions.** |  |  |
|  | *Location* | *- Does the time it takes to get there or distance of the place from your home determine where you take your child for treatment when (s)he is sick? If yes, why? If no, why not?*  *-Does transport fare determine what treatment and WHERE you seek care for a sick child?* |
|  | *Finances/ Out of Pocket Expenses* | *How much money did you spend on the things you did for the sick child (probe for amount of money spent on the home remedies used, new foods or drinks introduced because of the illness, other in-kind payments that had to be made relating to making the child better)*  *-Does cost of services determine WHERE you seek care for a sick child? How about the ease of paying (e.g. buying on credit, paying small-small) for services determine WHERE you seek care for a sick child?*  *-Does cost determine WHEN you seek care for a child?*  *- Do you consider your role to pay for the care”? (probe for reasons why the care giver does not consider it his/her role e.g. culture, gender roles, finances, religion)* |
| **Objective 4: Understand how previous experiences and perceptions of quality of care across types of providers affect care seeking patterns for sick children.** | *Perceptions of Quality of Care*  *(related to the last illness episode)* |  |
|  | *Previous experiences with different providers*  *(not related to the last previous illness episode)* | *-* *Tell me about your experience seeking care for your child/ children. Which providers in this community have you visited?*  *Explore the following questions for each provider:*  *When you went to the provider for treatment, how did the provider respond?*  *Did the provider explain the illness?*  *Did the provider explain treatment?*  *Did the provider listen to your questions/ concerns?*  *Did the provider answer your questions/ concerns?*  *What treatments were provided if any? How did child/ children respond to the treatment?*  *- Did any previous experience (good/ bad) influence why you did not seek care?* |
| *Interviewer: Thank you for your answers in the previous section. Now let us talk about the things that happen in your community about seeking care for a sick child under-5* | | |
| **Objective 3: Explore how social norms, gender roles, and financial and geographic access influence treatment and care seeking decisions.** | *Barriers and facilitating factors related to the communities’ social norms and gender roles* | *- In this community, when a child is sick, how does a mother usually respond? Explain.*  *-How do community/religious leaders influence care seeking for a sick child?* |
| **Objective 2: Understand how women, men and family members interact in terms of seeking advice and negotiating access to care.** | *Social Norms* | *Are there any practices/ beliefs in your community that influences seeking advice for a sick child?*  *Are there any practices/ beliefs in your community that influences how you get access to care outside the house for a sick child?* |

**HEALTH PROVIDERS**

Interviewer: Thank you for agreeing to this visit and interview. This interview will be focused on understanding how parents in this community seek care for their children under 5 years old who are sick with fever, diarrhea and pneumonia.

Introduction of Respondent (Warm- Up): Can you introduce yourself?

| State |  |
| --- | --- |
| LGA |  |
| Community |  |
| Respondent Type |  |
| Respondent Code |  |
| Age |  |
| Interview Date |  |
| Start time |  |
| End Time |  |

| *Objectives* | *Themes* | *Questions* |
| --- | --- | --- |
| **Objective 1: Explore how presentation, recognition and interpretation of illness signs and symptoms in the household influence care seeking for sick children.** | *Presentation and Recognition of illness signs and symptoms* | *-What are the signs and symptoms that make the caregivers in this community come to you when a child under 5 has diarrhoea?*  *- What are the signs and symptoms that make the caregivers in this community come to you when a child under 5 has fever?*  *- What are the signs and symptoms in this community that make the caregivers come to you when a child under 5 has pneumonia?* |
|  | *Severity* | *For each of the illnesses, which of the signs/ symptoms do care givers usually demand your immediate attention?*  *Do you know any reasons why they consider the signs and symptoms as severe?* |
|  | *Causes* | *In your opinion, do the people in this community know what causes fever in children?*  *-In your opinion, do the people in this community know what causes pneumonia in children?*  *-In your opinion, do the people in this community know what causes diarrhea in children?* |
|  | *Treatment* | *-When a child is brought to you with diarrhoea, do you do anything about the symptoms? (Probe for what is done; treatments given) Why do you use this treatment?*  *-When a child is brought to you with pneumonia, do you do anything about the symptoms? (Probe for what is done; treatments given)? Why do you use this treatment?*  *-When a child comes to you with fever, do you do anything about the symptoms? (Probe for what is done; treatments given) Why do you use this treatment?* |
|  | *Timing of care* |  |
| **Objective 2: Understand how women, men and family members interact in terms of seeking advice and negotiating access to care.** | *Household gender roles related to childcare* | *-Do fathers ever bring sick children for care in this community? Why, or why not?*  *Do mothers and fathers ever come together with their sick children for care in this community? Why, or why not?* |
|  | *Social Norms* | *-What are some of the reasons a sick child may not be taken for care in this community? (probe for: malnourished child, child with handicap…)*  *-Is there a difference in how a sick female child is cared for, compared to a sick male child? What are the differences? (Probe for difference between birth order or any other reason)* |
|  | *Decision-making conversations* |  |
|  | *Negotiating Access to care* |  |
| **Objective 3: Explore how social norms, gender roles, and financial and geographic access influence treatment and care seeking decisions.** | *Barriers and facilitating factors related to the communities’ social norms and gender roles* | -*What role do community/religious leaders play in influencing cultural practices/ beliefs related to seeking care for a sick child?*  *-What should be done to improve the child healthcare seeking practices in this community?* |
|  | *Location* | *-Do you think travel distance determines the parents’ choice of healthcare provider for a sick child? explain*  *- Do you think travel time determines the parents’ choice of healthcare provider for a sick child? explain* |
|  | *Finances/ OOPE* | *- Do you think travel costs determines the parents’ choice of healthcare provider for a sick child? explain*  *-Does the cost of paying for services determine the parents’ choice of healthcare provider for a sick child? Explain*  *- What do the parents need to pay for when they bring their children to seek care? (Give a list of broad areas and average prices e.g. registration, tests, drugs and injections etc. for the 3 illnesses)*  *-* *Are parents asked to bring or go and buy any materials/supplies when seeking care for their child from you?*  *-How do you respond if a parent arrives with a sick child but is unable to pay?* |
| **Objective 4: Understand how previous experiences and perceptions of quality of care across types of providers affect care seeking patterns for sick children.** | *Perceptions of Quality of Care* | *- What do you consider to be quality health service? (probe for helpfulness of health provider, outcome of care, waiting time, ability of health providers to answer questions, attitude of the health provider, ability of the health provider to understand and respond to specific need)*  -What are the things you will do that will make you say that the service you provided is of good (quality)?  *Probes:*  *is it how helpful you are to the patient?*  *Is it determined what happens to the child?*  *Is it determined by how long the patient had to wait before you attended to them?*  *Is it determined by how well the you are able to answer care givers question?*  *Is it determined by how you treat the care givers?*  *Is it determined by how well the you understood and considered specific things the care giver asked for*  *So from your explanation above, Are there circumstances in which you are not able to provide quality healthcare for sick children in this community? Explain*  *-What factors would enable you to provide quality healthcare for sick children in your community? (probe for issues of workload, space)* |
| *(Add probe across board to avoid getting yes/ no answers)* | *Previous experiences with different providers* | *-Do you think that a care giver’s experience with a health provider or other staff in the facility in the past affects whether or not s/he seeks health care when ill? Have care givers shared concerns about the quality of care they have received in the past with you?* |
|  | *Providers perceived role in providing care* | *Tell me about the last time you provided care for a sick child brought in by a care-giver*  *-Probes:*  *When it occurred*  *Where the care-seeking conversations with parents of sick children took place?*  *Which family members participated in these conversations?*  *-How long did the conversations take?*  *-What information was discussed?*  *-Did the parents ask any questions? If so, what questions did they ask? How did you respond?*  *-* *What role can you play in improving health seeking behavior of parents for their children in this community? (probe for counselling on recognition of danger signs, timely care-seeking, appropriate care-seeking)*  *-Are there community-based health groups in your community?*  *-If yes, what is their function and impact?*  *-If yes, how do you work with them to encourage child healthcare seeking?* |

**COMMUNITY LEADERS**

Introduction/ Warm-Up: Thank you for agreeing to this visit and interview. This interview will be focused on understanding how parents in this community seek care for their children under 5 years old who are sick with fever, diarrhea and pneumonia.

| State |  |
| --- | --- |
| LGA |  |
| Community |  |
| Interview Date |  |
| Start time |  |
| End Time |  |
| Gender |  |
| Age |  |
| Level of Education |  |
| Occupation |  |
| Number of Children |  |

| *Objectives* | *Themes* | *Questions* |
| --- | --- | --- |
| **Objective 2: Understand how women, men and family members interact in terms of seeking advice and negotiating access to care.** | *Household gender roles related to childcare* | *- Who is responsible for caring for children less than five years - in this community?*  *- Who is responsible for caring for these children when they get sick?*  *-What usually happens when a child is sick in this community?*  ***(****Probe for* *home care traditional methods – praying for health…)*  *-* *In this community, what are the roles of other family members in caring for a sick child? (Probe for grandfather, grandmother, uncle, aunty)* |
|  | *Social Norms* | *What are the practices/ beliefs in your community that influences seeking advice for a sick child?*  *What are the practices/ beliefs in your community that influences how you get access to care outside the house for a sick child?* |
|  | *Decision-making conversations* | *- In this community, who do mothers talk to when a child under 5 is sick?*  *-In this community, who do fathers talk to when a child under 5 is sick?*  -*Are others in the household or community involved in discussions and decisions to seek advice or care outside the home? Please explain*  ***Probe for***  *🡺 whether Community or religious leaders are involved in the discussions*  *🡺 who initiates the discussion?*  *🡺 who takes part in the discussion?*  *🡺 what is typically discussed?*  *-How long after the illness begins do these conversations typically take place?*  *-What are these conversations about?*  ***Probe for***  *🡺 type of care selected?*  *🡺 cost of treatment?*  *🡺 transport?*  *🡺 type of illness/diagnosis?*  *🡺 severity?* |
|  | *Negotiating Access to care* | *- How does a mother seek care for a sick child? (Probe for permission, finances, determining when and where to seek care)*  *-How does a father seek care for a sick child? (Probe for permission, finances, determining when and where to seek care)*  *-* *In this community, does a mother have to get permission to seek care for a sick child outside the home?*  ***Probes***  *🡺If yes, from who?*  *🡺What are some of the reasons permission may be granted, or may not be granted?*  *🡺If permission is not granted, what can be done?*  *-Who makes the [final?] decision to seek care for a sick child? Why? Who decides when and where advice and/or treatment is sought?* |
| **Objective 3: Explore how social norms, gender roles, and financial and geographic access influence treatment and care seeking decisions.** | *Barriers and facilitating factors related to the communities’ social norms and gender roles* | *-What practices/ beliefs in your community influence if and where to seek care when a child is ill?*  *-Do community/religious leaders influence whether or not to seek care for a sick child, or what kind of care is sought? If yes, how?*  *-Does the type of illness or severity of illness influence when and where advice is sought?* |
|  | *Location* | *Does the time it takes to get there or distance of the place from your home determine where people in this community take their sick children for treatment? If yes, why? If no, why not?*  *-Does transport fare determine what treatment and WHERE you seek care for a sick child?* |
|  | *Finances/ OOPE* | *- Does the cost of services determine where people in this community seek care for a sick child?*  *- What kinds of financial support is available from the community to assist families with a sick child?*  *Does the cost of care influence who can make the decision on choice of health care provider?* |
